# Delivery of miR-200c-3p Using Tumor-Targeted Mesoporous Silica Nanoparticles for Breast Cancer Therapy

**DOI:** 10.1021/acsami.3c07541

**Published:** 2023-08-07

**Authors:** Iris Garrido-Cano, Anna Adam-Artigues, Ana Lameirinhas, Juan F. Blandez, Vicente Candela-Noguera, Ana Lluch, Begoña Bermejo, Felix Sancenón, Juan Miguel Cejalvo, Ramón Martínez-Máñez, Pilar Eroles

**Affiliations:** † Biomedical Research Institute INCLIVA, Valencia 46010, Spain; ‡ Instituto Interuniversitario de Investigación de Reconocimiento Molecular y Desarrollo Tecnológico (IDM), Universitat Politècnica de València, Universitat de València, Valencia 46010, Spain; § CIBER de Bioingeniería, Biomateriales y Nanomedicina (CIBER-BBN), Madrid 28029, Spain; ∥ Unidad Mixta de Investigación en Nanomedicina y Sensores, Universitat Politècnica de València, IIS La Fe, Valencia 46026, Spain; ⊥ Centro de Investigación Biomédica en Red de Cáncer (CIBERONC), Madrid 28029, Spain; # Universitat de València, Valencia 46010, Spain; ∇ Clinical Oncology Department, Hospital Clínico Universitario de Valencia, Valencia 46010, Spain; ○ Unidad Mixta UPV-CIPF de Investigación en Mecanismos de Enfermedades y Nanomedicina. Universitat Politècnica de Valencia, Centro de Investigación Príncipe Felipe, Valencia 46012, Spain

**Keywords:** mesoporous silica nanoparticles, breast cancer, microRNA, therapy, targeted delivery

## Abstract

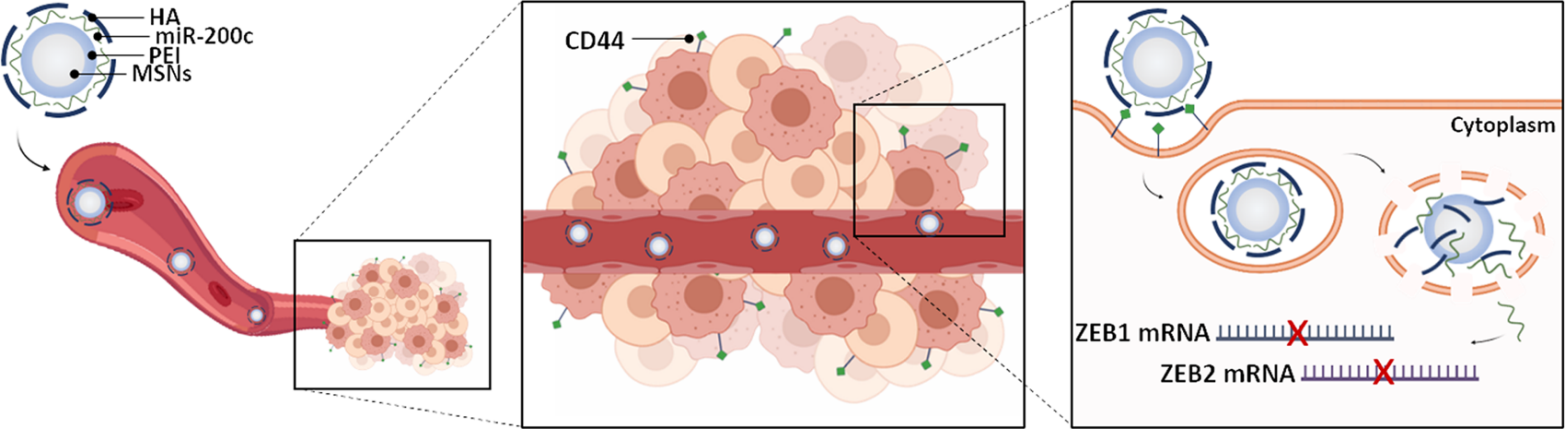

Despite advances in breast cancer treatment, it remains the leading cause of cancer-related death in women worldwide. In this context, microRNAs have emerged as potential therapeutic targets but still present some limitations for *in vivo* applications. Particularly, miR-200c-3p is a well-known tumor suppressor microRNA that inhibits tumor progression and metastasis in breast cancer through downregulating *ZEB1* and *ZEB2*. Based on the above, we describe the design and validation of a nanodevice using mesoporous silica nanoparticles for miR-200c-3p delivery for breast cancer treatment. We demonstrate the biocompatibility of the synthesized nanodevices as well as their ability to escape from endosomes/lysosomes and inhibit tumorigenesis, invasion, migration, and proliferation of tumor cells *in vitro*. Moreover, tumor targeting and effective delivery of miR-200c-3p from the nanoparticles *in vivo* are confirmed in an orthotopic breast cancer mouse model, and the therapeutic efficacy is also evidenced by a decrease in tumor size and lung metastasis, while showing no signs of toxicity. Overall, our results provide evidence that miR-200c-3p-loaded nanoparticles are a potential strategy for breast cancer therapy and a safe and effective system for tumor-targeted delivery of microRNAs.

## Introduction

Breast cancer (BC) is the most prevalent type of cancer and the leading cause of cancer-related death among women worldwide.^1^ Despite advances in treatment, a significant number of BC patients develop therapeutic resistance, which is one of the major concerns in clinics. As a consequence, around 20–30% of patients relapse with metastatic disease. At this stage, the 5-year survival rate is approximately 25%.^2,3^ Therefore, new therapeutic strategies are needed to improve patient outcomes. Particularly, microRNAs (miRNAs) have emerged as a promising therapeutic strategy for BC treatment.^4^

miRNAs are small single-stranded noncoding ribonucleic acids (RNAs) of 19–25 nucleotides in length that regulate the expression of numerous target mRNAs.^5^ Hence, miRNAs are involved in numerous physiological processes, such as cell development and differentiation, and are also enrolled in pathological processes, such as inflammatory diseases and cancer. In the context of cancer, abnormal expression of miRNAs is usually found in tumors, leading to deregulation of target genes involved in hallmarks of cancer such as cell proliferation, invasion, apoptosis, or drug resistance.^6−8^

miR-200c is found among the most well-described tumor suppressor miRNAs in BC. It is a member of the miR-200 family, which is a widely known family of tumor suppressor miRNAs that inhibits the expression of *ZEB1* and *ZEB2*, which are master regulators of the epithelial to mesenchymal transition (EMT), involved in tumor progression, drug resistance, and metastasis.^9,10^ Accordingly, miR-200c has been reported to be downregulated in metastases and also within the primary tumors, and its overexpression decreases the capacity of tumor cells to undergo EMT, invade, and metastasize.^9^ Furthermore, miR-200c has been found downregulated in breast cancer stem cells (BCSCs), which is a subpopulation of tumoral cells that present innate drug resistance and have a significant important implication in disease relapse.^11−13^ In this context, EMT has been widely described to confer stem cell-like properties.^11,12^ In accordance with this, overexpression of miR-200c in BCSCs has been described to reduce tumorigenesis and tumor cell growth while decreasing their stem-like properties and promoting an epithelial-like phenotype. Therefore, miR-200c may be a potential therapeutic target in BC.

Despite their potential, the use of miRNAs *in vivo* presents some challenges, such as their instability due to rapid degradation by cellular and serum nucleases and their low ability to cross cell membranes because of their small size and negative charge. Thus, the delivery in cells remains the most significant hurdle in using miRNAs as therapeutic agents.^14,15^ In this scenario, nanoparticles are potential tools for miRNA pharmacokinetics improvement, since they can avoid miRNA degradation, can ensure their delivery to the cytosol,^16^ accumulate in tumors thanks to the enhanced permeability and retention (EPR) effect,^17^ and could additionally be functionalized to target specific cells.^18,19^ In particular, mesoporous silica nanoparticles (MSNs) are suitable candidates as miRNA delivery vectors.^20^ MSNs are biocompatible and biodegradable^21,22^ materials with a porous structure, large specific surface area and volume, chemically and thermally stable, and can be equipped with molecular gates (also known as gatekeepers or nanovalves) able to regulate cargo delivery in response to predefined physical, chemical, or biochemical stimuli.^23−27^ Thanks to their advantageous properties, MSNs have become one of the most important platforms for drug-controlled release^28,29^ and they have been used in many biomedical applications, such as drug,^30,31^ gene,^32,33^ and RNA^34,35^ delivery; bioimaging;^36,37^ chemical communication;^38,39^ stigmergy;^40,41^ nanomotors;^42,43^ biosensing;^44,45^ and theragnostic^46^ and tissue engineering.^47,48^ MSNs can be functionalized not only with molecular gates but also with other organic ligands and inorganic nanoparticles to provide them versatile chemical and physical properties,^49,50^ which made them extraordinary versatile nanoplatforms.

The aim of this work is to develop a new therapeutic strategy for BC based on MSNs loaded with miR-200c-3p and to test its efficiency as a targeted transfection vector and tolerability *in vitro* and *in vivo*. Based on this, we report herein the preparation of MSNs containing miR-200c-3p, PEI, and HA (MSN-PEI-miR200c-HA) for the targeted delivery of miRNA in BC tumors. *In vitro* studies were carried out to demonstrate the capability of the nanocarrier to target the CD44 receptor, their biocompatibility, and their ability to escape from endosomes and lysosomes, which is crucial to ensure the miRNA’s functionality. Besides, we demonstrate that the synthesized nanodevices revert EMT and stem-like phenotype, and inhibit invasion, migration, tumorigenic ability, and proliferation of tumor cells. Besides, the nanoparticles were also tested *in vivo* in a triple-negative BC (TNBC) orthotopic xenograft mouse model, where a remarkable tumor growth reduction and metastasis inhibition were observed.

## Methods

### Chemical Reagents

*n*-Cetyltrimethylammonium bromide (CTABr; purity ≥98%), tetraethyl orthosilicate (TEOS; purity: 98%), sodium hydroxide (NaOH; purity ≥98%), polyethylenimine (PEI; average molecular weight: 25 000 g/mol), hyaluronic acid (HA; #53747), propidium iodide (PI; purity ≥94%), diamidino-2-phenylindole dihydrochloride (DAPI; purity ≥98%), lysosome Isolation Kit (LYSISO1), and tetramethylammonium hydroxide solution (TMAH: 25 wt % in H_2_O) were purchased from Sigma-Aldrich (St. Louis, MO). Tetramethylrhodamine-5-isothiocyanate (TRITC; T490), RNA labeled with Cy3 (miRCy3, AM4621), DMEM/F12, RPMI 1640, l-glutamine, penicillin-streptomycin (10000 U/mL), fetal bovine serum (FBS), formaldehyde (28908), LysoTracker Deep Red (L12492), Hoechst33342, scramble miRNA (4464058), and Lipofectamine 2000 were acquired from Thermo Fisher Scientific (Waltham, MA). miR-200c-3p was acquired from Guangzhou RiboBio CO (Guangzhou, China).

### Synthesis of MSNs

MSNs were synthesized as previously described.^51^ Briefly, CTABr was used as the structure-directing agent. One g (2.74 × 10^–3^ mol) was dissolved in 480 mL of deionized water, and aqueous NaOH (3.5 mL, 2 M in deionized water) was added. The temperature was then adjusted to 80 °C, and the silica source TEOS (5 mL, 2.57 × 10^–2^ mol) was added dropwise while the solution was being vigorously stirred. The mixture was stirred for 2 h to give white a precipitate (as-synthesized MSNs), which was collected by centrifugation (9500 *g*, 20 min), washed with deionized water until reaching neutral pH, and dried at 60 °C overnight. As-synthesized MSNs were calcined at 550 °C using an oxidant atmosphere for 5 h to remove the template phase, thus obtaining the surfactant-free final MSNs.

### Synthesis of MSN-PEI-miR200c-HA and MSN-PEI-miRCy3-HA

70 mg of MSNs was resuspended in a mixture of 1.7 mL of PBS 41× and 25 mg of PEI (*M*_w_ = 25 kDa) and stirred for 3 h at room temperature. After that, solids were centrifuged (9500*g*, 10 min), resuspended in a water solution (3.5 mL, 10 μM) of either miR-200c-3p (for MSN-PEI-miR200c-HA) or miRCy3 (for MSN-PEI-miRCy3-HA), and stirred for 30 min at room temperature. Then, a water suspension of HA was added (0.5 mg/mL, 3.5 mL) and stirred for 3 h at room temperature. The resulting solid was recovered by centrifugation (9500*g*, 10 min), washed several times with water to remove the excess HA, and dried. Control nanoparticles (MSN-PEI-HA) were obtained by following the same procedure without the addition of miR-200c-3p.

### Synthesis of MSN-TRITC-PEI-miR200c-HA

35 mg of MSN was resuspended in 3 mL of anhydrous ethanol and 4.48 μmol of tetramethylrhodamine (TRITC) and stirred at room temperature overnight. Then, the suspension was centrifuged (9500*g*, 10 min) and dried under vacuum. Next, solids were resuspended in a mixture of 0.85 mL of PBS 41×, and 12.5 mg of PEI (*M*_w_ = 25 kDa), and stirred for 3 h at room temperature. After that, solids were centrifuged (9500*g*, 10 min), resuspended in a water solution of miR-200c-3p (1.75 mL, 10 μM), and stirred for 30 min at room temperature. Then, a water suspension of HA was added (0.5 mg/mL, 1.75 mL) and stirred for 3 h at room temperature. The resulting solid was recovered by centrifugation (9500*g*, 10 min), washed several times with water to remove the excess HA, and dried.

### Characterization of Materials

The prepared materials were analyzed by using standard techniques. Powder X-ray diffraction (PXRD) patterns were obtained with a Philips D8 Advance (Philips, Amsterdam, The Netherlands) diffractometer using Cu Kα radiation. Transmission electron microscopy (TEM) images were taken by a JEOL JEM-1010 (JEOL Europe SAS, Croissysur-Seine, France), working at 100 kV and TEM-energy dispersive X-ray (EDX) analysis was performed in a JEOL JEM-2100F (JEOL Europe SAS) working at 200 kV. N2 adsorption–desorption isotherms were recorded with a Micromeritics ASAP 2010 automated desorption analyzer (Micromeritics Instrument Corporation, Norcross). Samples were degassed at 120 °C in a vacuum for 24 h. The specific surface areas were determined from the adsorption data in the low-pressure range by using the Brunauer, Emmett, and Teller (BET) model. Pore size was determined by following the Barret, Joyner, and Halenda (BJH) method. Dynamic light scattering (DLS) and ζ potential measurements were conducted with a Malvern Zetasizer Nano ZS. Fourier transform infrared (FTIR) was recorded on a Vertex 70 V FTIR spectrometer (Bruker, Billerica, Massachusetts) in the range of 4000 to 500 cm^–1^. Thermogravimetry of the materials was performed using a STARe System TGA/DSC 3+ from Mettler Toledo (Mettler Toledo, Inc., Schwarzenbach, Switzerland). Loss weight in an oxidant atmosphere (air, 80 mL·min^–1^) was registered within a dynamic step in which was applied an increase of 10 °C min^–1^ in the interval from 20 to 1000 °C, with an isotherm for 1 h at 100 °C.

### Cell Culture

MDA-MB-231, 4T1, and OE19 cells were obtained from the American Type Culture Collection (ATCC, Virginia). MDA-MB-231 cells were cultured in DMEM/F12 supplemented with 10% (v/v) FBS and 1% (v/v) penicillin-streptomycin (10,000 U/mL). OE19 were cultured in RPMI 1640 supplemented with 10% (v/v) FBS, 1% l-glutamine, and 1% (v/v) penicillin-streptomycin (10,000 U/mL). All cell lines were maintained at 37 °C in a humidified atmosphere containing 5% CO_2_.

### Apoptosis Analysis

To study the toxic effect of MSN-PEI-miR200c-HA, MDA-MB-231 cells were seeded in 96-well plates at 8 × 10^3^ cells per well. After 24 h, the cells were treated with nanoparticles (1, 10, 100 μg/mL) and incubated for 72 h. Then, the cells were collected and stained with annexin V-FITC (Immunostep, Salamanca, Spain) and PI (20 μg/mL) in annexin V binding buffer (Immunostep) for 15 min at room temperature, and the cells were analyzed using BD LSRFortessa X-20 (BD Biosciences, NJ). Data were processed using a FlowJo V10 (FlowJo, LLC).

### Hemolysis Assay

Blood from BALB/C nude mice was collected in EDTA tubes and centrifuged at 500*g* for 5 min to isolate the red blood cells (RBC), which were washed three times with PBS and resuspended in the same buffer (hematocrit 3%). Twenty μL of nanoparticles at different concentrations dissolved in PBS was added to 180 μL of RBC suspensions to reach the final concentrations of 0.1, 1, 10, and 100 μg/mL of MSN-PEI-miR200c-HA. The mixture was incubated for 1 h at 37 °C. Then, samples were centrifuged at 500xg for 5 min, and the absorbance of the supernatant was measured at 540 nm by using the microplate reader Spectra Max Plus (Molecular Devices, San José, California) to determine hemoglobin release. PBS and Triton X-100 (1% (v/v)) were used as negative and positive controls, respectively. Percentage of hemolysis was calculated as [(Sample absorbance – negative control)/(positive control – negative control) × 100].

### Nanoparticles’ Internalization

5 × 10^4^ MDA-MB-231 and OE19 cells (5 × 10^4^ cells) were seeded in cover glass slides (Ibidi GMBH, Germany). After 24 h, the cells were treated with MSN-TRITC-PEI-miR200c-HA or MSN-PEI-miRCy3-HA (25 μg/mL) for 15 min, washed with PBS, and fixed with a solution of 4% formaldehyde and DAPI (4′,6-diamidino-2-phenylindole, 5 μg/mL). Cellular uptake was evaluated by confocal microscopy (TCS SP2, Leica Biosystems, Germany). The red mean fluorescence intensity (MFI) deriving from TRITC was determined in a minimum of 200 cells using ImageJ software (ImageJ 1.51h, NIH) and referenced to MDA-MB-231.

### CD44 Expression Determination by Flow Cytometry

1 ×10^6^ MDA-MB-231 and OE19 cells were incubated with a phycoerythrin-conjugated monoclonal antibody against human CD44 (550989, BD Biosciences) according to the manufacturer’s instructions for 30 min. An isotype control (555749, BD Biosciences, New Jersey) was used as a negative control. The cells were washed and analyzed using a flow cytometer BD LSRFortessa (BD Biosciences). Analysis was carried out with FlowJo V10 software (BD Biosciences).

### miRNA Release Assay

Release assays were performed in PBS, acetate buffer, and lysosomal extract (obtained from rabbit liver by using LYSISO1 following the manufacturer’s instructions). In summary, nanoparticles were suspended in deionized water (0.5 mg mL^–1^). After that, 1 mL of the suspension was added to two Eppendorf tubes and stirred at 37 °C. An aliquot in each tube was taken and centrifuged to consider the time 0. Then, 0.8 mL of PBS or 0.8 mL of lysosomal extract was added to the tubes, and different aliquots were taken and centrifuged over time. The supernatants of aliquots were analyzed to monitor the release of miRCy3 by determining fluorescence (λ_ex_ = 547 nm, λ_em_ = 562 nm) in a Jasco FP-8500 spectrofluorometer (Jasco Analitica Spain, Madrid, Spain).

### Lysosomal Escape Evaluation

MDA-MB-231 cells (5 × 10^4^) were seeded in cover glass slides (Ibidi GmbH, Gräfelfing, Germany). After 24 h, the cells were incubated with LysoTracker Deep Red and Hoechst33342 for 1 h following the manufacturer’s protocol and treated with 25 μg/mL MSN-PEI-miRCy3-HA for different time periods. Then, the cells were washed with PBS and visualized by confocal microscopy (TCS SP8, Leica). Colocalization analysis between MSN-PEI-miRCy3-HA and endosomes/lysosomes was carried out using the ImageJ JACoP plugin.^52^ Threshold value was set to minimize background signal, and the fraction of overlapping intracellular pixels was calculated by Manders’ coefficient considering the threshold coefficient of colocalization of MSN-PEI-miRCy3-HA with endosomes/lysosomes. A minimum of 150 cells per condition were analyzed.

### Cell Transfection

MDA-MB-231 cells were transfected with miR-200c-3p or scramble miRNA at 50 nmol/L using Lipofectamine 2000 following the manufacturer’s instructions.

### Quantitative Real-Time PCR (qRT-PCR)

Total RNA was extracted from cells or frozen tissue samples using Trizol (Invitrogen, Massachusetts) reagent, following the manufacturer’s instructions. cDNA was synthesized by reverse transcription using a TaqMan MicroRNA Reverse Transcription Kit (Thermo Fisher Scientific) for miRNAs, or High-Capacity cDNA Reverse Transcription Kit (Thermo Fisher Scientific) for mRNAs. qRT-PCR was performed using TaqMan assays (Thermo Fisher Scientific). miR-200c-3p (ID: 002300) expression was calculated relative to the RNU43 (ID: 001095) expression. To evaluate the effect of nanoparticles in metastasis, human *HPRT* (*hHPRT*, Hs02800695_m1) expression was calculated relative to mouse *gapdh* (mgapdh, Mm99999915_g1).^53,54^ Relative expression was calculated by using the 2^–ΔΔCt^ method.

### Western Blot

Protein was isolated from cells or frozen tumor samples using RIPA buffer with protease and phosphatase inhibitors (Thermo Fisher Scientific). Proteins were separated by SDS-PAGE and transferred to a nitrocellulose membrane (Bio-Rad, CA). After blocking, membranes were incubated with primary antibodies to CD44 (3570, Cell Signaling, Massachusetts), ZEB1 (3396, Cell Signaling), ZEB2 (ab138222, Abcam, Cambridge, U.K.), E-cadherin (610181, BD Biosciences), N-cadherin (4061, Cell Signaling), Fibronectin (ab32419, Abcam), β-catenin (9562, Cell Signaling), Vimentin (550513, BD Biosciences), Nanog (4903, Cell Signaling), Oct-4A (2840, Cell Signaling), Sox2 (3579, Cell Signaling) or GAPDH (AM4300, Thermo Fisher Scientific), and secondary antibodies antimouse (7076, Cell Signaling) or antirabbit (7074, Cell Signaling). Proteins were detected using a Pierce ECL Western Blotting Substrate (Thermo Fisher Scientific) in ImageQuant Las 4000 (GE Healthcare, IL).

### Cell Proliferation Assay

3 ×10^3^ MDA-MB-231 cells were seeded into 96-well plates. After 24 h, the cells were treated with MSN-PEI-HA or MSN-PEI-miR200c-HA (20 μg/mL). Untreated cells were included as controls. Cell proliferation was assessed at 0, 24, 48, 72, and 120 h by using WST-1 reagent (Sigma-Aldrich) following the manufacturer’s protocol.

### Cell Cycle Analysis

MDA-MB-231 cells were treated with MSN-PEI-HA or MSN-PEI-miR200c-HA (20 μg/mL). Untreated cells were included as a control. After 72 h, the cells were collected by trypsinization, washed with PBS, and fixed in cold 70% ethanol at −20 °C overnight. Afterward, the cells were incubated with Propidium iodide/RNase staining solution (Immunostep, Salamanca, Spain) overnight at 4 °C and analyzed on BD LSRFortessa. Analysis was carried out using ModFit LT software (Verity Software House, ME).

### Transwell Invasion and Migration Assays

MDA-MB-231 cells were treated with MSN-PEI-HA or MSN-PEI-miR200c-HA (20 μg/mL) for 72 h, and 5 × 10^4^ cells were seeded in the upper chamber of 24-well inserts (8 μm pore size) (Millipore, Massachusetts) with FBS-free medium. The lower chamber was filled with complete medium. For invasion assays, the upper chamber was previously coated with matrigel (Corning, NY). The cells were allowed to migrate or invade for 24 h, prior to fixation with methanol and staining with crystal violet. The cells remaining on the upper surface of the membrane were removed with a cotton swab. Number of cells was analyzed by using ImageJ software.

### Colony Formation Assay

MDA-MB-231 cells were treated with MSN-PEI-HA or MSN-PEI-miR200c-HA (20 μg/mL) for 72 h. Then, 500 cells per well were seeded in six-well plates. After 10 days, the cells were fixed with methanol and stained with crystal violet. The colonies that formed with more than 50 cells were counted under a microscope.

### *In Vivo* Experiments

Six-week-old female BALB/C nude mice were acquired from Charles River Laboratories (Massachusetts). 1.6 × 10^6^ MDA-MB-231 cells in 100 μL of Matrigel/PBS (1:1) were injected into the mammary fat pad of the mice (*N* = 27). Once tumors were palpable, mice were randomly divided into 3 groups of 9 animals and treated separately with PBS, MSN-PEI-HA, or MSN**-**PEI-miR200c-HA. Treatments were administered intravenously two times per week for 28 days. Nanoparticles were administered at 10 mg/kg. Tumors were measured two times per week and tumor volume was calculated using the following formula: (shortest diameter)^2^ × (longest diameter) × 0.5. All experimental protocols involving animals were approved by the Institutional Review Board of INCLIVA (2020/VSC/PEA/0131). Animals were sacrificed at the end point or when they met the institutional euthanasia criteria for tumor size or overall health condition.

### Serum Analysis

Blood was immediately collected after sacrifice, allowed to clot for 30 min at room temperature, and centrifuged at 10,000 rpm for 15 min at 4 °C. Serum was isolated and stored at −80 °C for biochemical analysis. Aspartate aminotransferase (AST), alanine transaminase (ALT), creatinine (CRE), and urea (URE) were determined.

### Silicon Biodistribution Analysis

48 h after the last treatment, animals were sacrificed. The tumor and selected organs (lungs, kidney, liver, and spleen) were collected and stored at −80 °C for silicon (Si) detection. Tissues were weighed and digestion was carried out into 1 mL of TMAH (25% (v:v) in water) in polytetrafluoroethylene (PTFE) vials at 80 °C for 2 h in a digestion unit Bloc digest 20 (Selecta, Barcelona, Spain). After cooling, samples were diluted in Milli-Q water (1:10), filtered with 0.45 μm filters (17463443, Scharlab, Barcelona, Spain), and kept in polystyrene tubes. Si determination was performed by inductively coupled plasma mass spectroscopy (ICP-MS) in an Agilent 7900 instrument in H2 mode.

### Immunohistochemistry

Automated immunohistochemical staining was performed using VENTANA BenchMark XT (Roche, Basel, Switzerland) with UltraView Universal DAB Detection Kit (0526980600, Roche) and *K*_i_-67 antibody (790-4286, Roche).

### Statistical Analyses

All statistical analyses were performed by using GraphPad Prism 6.0. Student’s *t* test was conducted to compare the two groups. Data in abnormal distribution were analyzed by the Mann–Whitney *U* Test. Two-way ANOVA with post hoc Bonferroni correction was used for *in vivo* statistical analysis.

## Results and Discussion

### Design and Synthesis of Nanodevices

**Figure 1:**
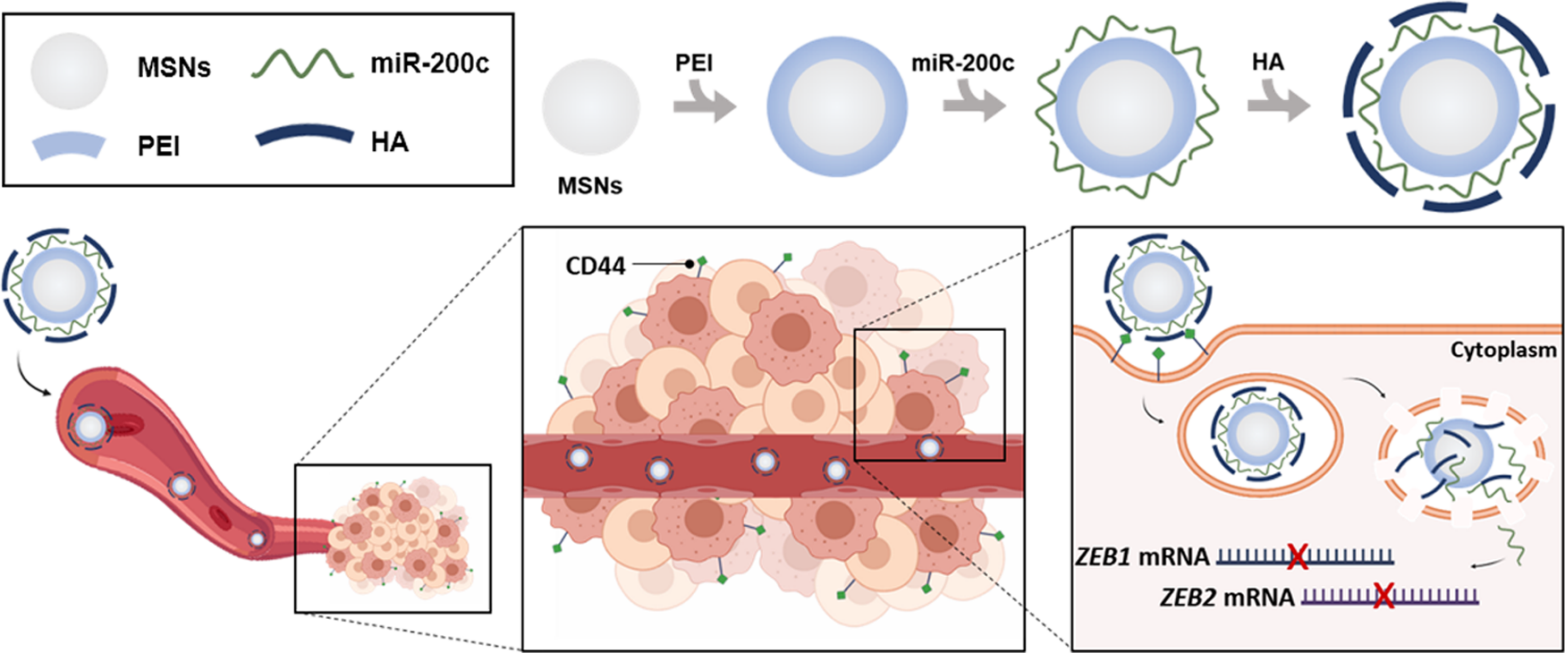
Schematic representation of the synthesis and mechanism of the MSN-PEI-miR200c-HA delivery system. After intravenous injection, MSN-PEI-miR200c-HA preferentially accumulates in tumors by the EPR effect, where HA actively targets the CD44 receptor in tumor cells and induces internalization. Then, PEI mediates lysosomal escape, and miR-200c-3p is released into the cytoplasm, where it inhibits the expression of *ZEB1* and *ZEB2*. MSNs: Mesoporous silica nanoparticles; PEI: polyethylenimine; miR-200c: miR-200c-3p; HA: hyaluronic acid.

Final nanodevices were prepared by following a layer-by-layer procedure on MSNs. The final nanodevice contains a core of MSN, a first layer of PEI followed by layers of miR-200c-3p and HA (MSN-PEI-miR200c-HA) (Figure 1). PEI was attached to the silanolate surface of MSN by electrostatic interactions due to its positive charge (amine groups). HA and miR-200c-3p were bound to MSN-PEI also electrostatically, given their negative charge (carboxy group in the case of HA, and orthophosphoric group in the case of miR-200c-3p). A potential limitation of MSNs as carriers of miRNAs is the endosomal entrapment, where the miRNAs’ biological function is impaired because of degradation.^55^ To avoid this, PEI was included in the nanoparticles. PEI is protonated once exposed to acidic pH, inducing a “proton sponge” effect giving rise to the scape of the nanoparticles from endosomes/lysosomes to the cytoplasm.^56,57^ Furthermore, nanoparticles were coated with HA, which is a biocompatible molecule that reduces the adsorption of proteins to the surface of nanodevices and their immunogenicity. Moreover, HA targets the CD44 receptor, which is involved in tumor progression and is overexpressed in BCSCs.^58,59^ In addition to the active targeting, our nanoparticles can passively target tumors *in vivo* due to the EPR effect.^60,61^ In addition to MSN-PEI-miR200c-HA, a similar nanoparticle lacking miR-200c-3p was also prepared as a control (MSN-PEI-HA).

### Characterization of Nanodevices

**Figure 2:**
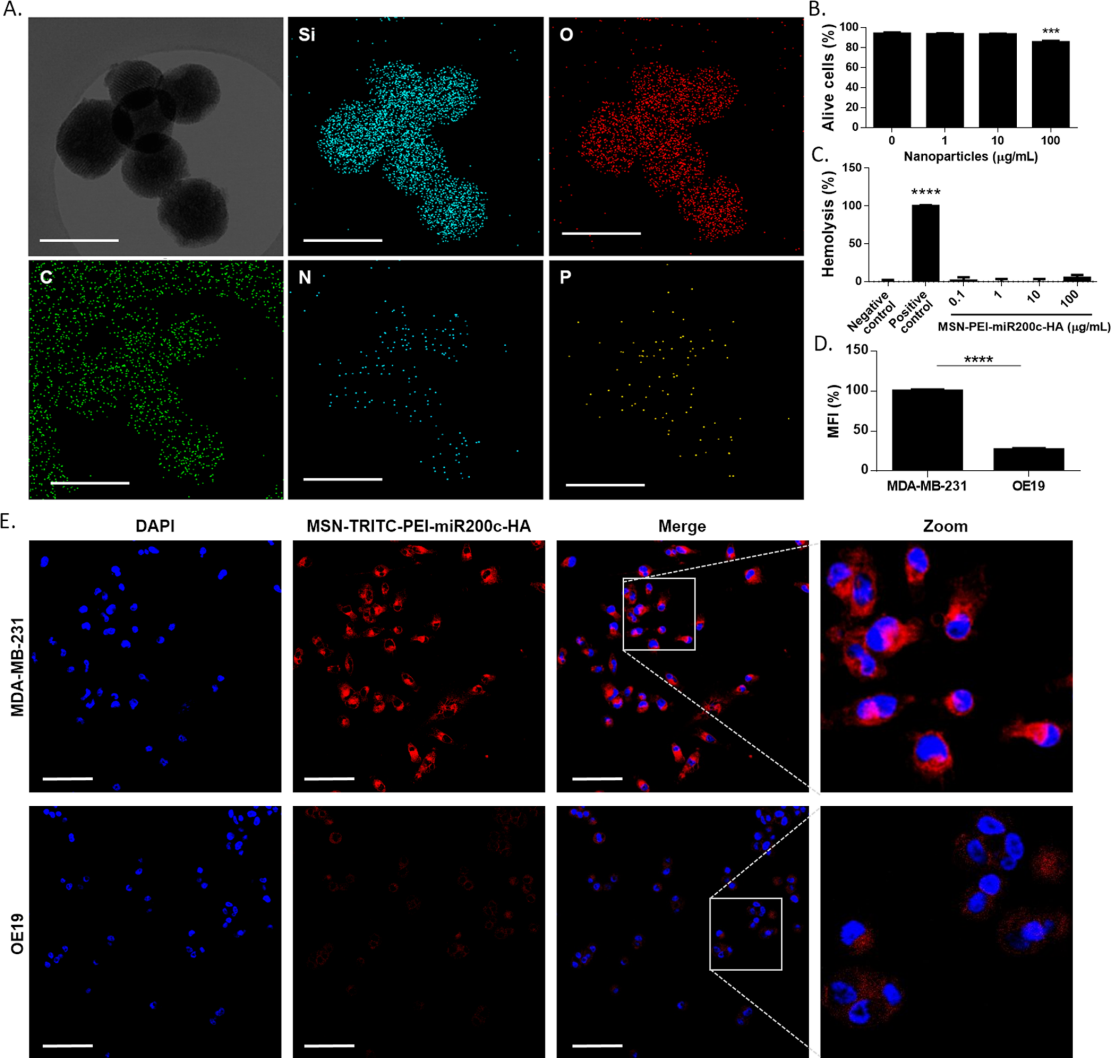
Characterization of MSN-PEI-miR200c-HA. (A) TEM images of MSN-PEI-miR200c-HA and EDX elemental mapping of Si, O, C, N, and P. Scale bar: 100 nm. (B) MDA-MB-231 cells were treated with MSN-PEI-miR200c-HA at different concentrations for 72 h and evaluated for apoptosis by flow cytometry after PI and FITC-Annexin V staining. Untreated cells were included as a control (mean ± SD). (C) Hemolytic activity of MSN-PEI-miR200c-HA. Red blood cells were incubated with PBS (negative control), 1% Triton X-100 (positive control), or MSN-PEI-miR200c-HA at different concentrations for 1 h at 37 °C. Percentage of hemolysis (mean ± SD). (D, E) Internalization of MSN-PEI-miR200c-HA in MDA-MB-231 and OE19 cell lines after 15 min of exposure to nanoparticles (25 μg/mL). Mean red fluorescent intensity (MFI, mean ± SEM) (D) and representative confocal images (scale bar: 75 μm) (E). ***p < 0.001; ****p < 0.0001.

The nanoparticles were characterized by using standard techniques. PXRD patterns of the MSNs before calcination show the typical four Bragg peaks that could be indexed as (1 0 0), (1 1 0), (2 0 0), and (2 1 0) using a hexagonal cell with an *a*_0_ cell parameter of 47 Å corresponding with a structure of hexagonally packed mesopores, typical of MSNs. After calcination, there was a displacement of the peaks toward higher angles because of the condensation of silanol groups during the calcination step, which caused a cell contraction of 5 Å (Supporting Figure S1). N_2_ adsorption–desorption isotherms of calcined MSNs were also recorded to obtain information about the surface area, pore size, and pore volume (Supporting Figure S2). A typical type IV isotherm for mesoporous solids was obtained containing a sharp adsorption step between *P*/*P*_0_ values of 0.2 and 0.4, due to nitrogen condensation inside the pores. It was calculated that a surface area of 1122 m^2^/g was obtained by applying the Brunauer–Emmett–Teller (BET) model, and the application of the Barrett–Joyner–Halenda (BJH) model resulted in a pore size centered at 2.5 nm and a pore volume of 0.92 cm^3^/g. The nanoparticles were also observed by TEM to confirm a spherical morphology and ordered pore structure (Supporting Figure S3). Loading and functionalization did not induce structural changes in the TEM images. Furthermore, TEM-EDX mapping of MSN-PEI-miR200c-HA showed the presence of Si and O associated with the silica structure; C and N related with the functionalization with PEI, miR-200c-3p, and HA; and P due to the presence of miR-200c-3p. TEM-EDX results of MSN-PEI-HA (Si: 31.6 ± 0.5, O: 44.3 ± 2.9, C: 21.6 ± 2.1, N: 2.4 ± 0.3, P: 0.0 ± 0.0 wt %) and MSN-PEI-miR200-HA (Si: 31.7 ± 0.9, O: 41.8 ± 1.3, C: 21.4 ± 2.3, N: 4.6 ± 0.2, P: 0.6 ± 0.1 wt %) also evidenced the presence of miR-200c-3p in MSN-PEI-miR200c-HA (0.6 wt % of P) (Figure 2A). Besides, the size of nanodevices was further confirmed by DLS. The hydrodynamic ratio increased from 96 ± 1 nm for calcined MSNs to 126 ± 2 nm for final MSN-PEI-miR200c-HA (114 ± 7 nm for MSN-PEI-HA). Moreover, the ζ potential of samples was also measured. Calcined MSNs presented a ζ potential value of −34 ± 2 mV, while the ζ potential values in MSN-PEI- miR200c-HA and MSN-PEI-HA were 34 ± 1 for both of them. In addition, thermogravimetric analyses of the different solid prepared allowed estimating that the attributable organic content of PEI, miRNA, and HA in MSN-PEI-miR200c-HA was 19.7, 0.7, and 1.3% of weight, respectively (Supporting Figure S4). FTIR studies were also carried out to monitor the synthesis of the nanoparticles (Supporting Figure S5). Thus, the combination of different characterization techniques confirmed the successful preparation of the nanodevices. Finally, we analyzed the stability of MSN-PEI-miR200-HA in different media. The nanoparticles were resuspended for 1 h and sonicated generously. We selected 1 h to simulate in vivo circulating time.^16,62^ We observed that the nanoparticles were stable in water (hydrodynamic diameter after 1 h: 131 nm, PDI: 0.301), yet the hydrodynamic diameter increased to ca. 400 nm after 1h when the nanoparticles were resuspended in DMEM + 10% of FBS, suggesting a partial aggregation or protein corona formation.

To study the potential toxicity of the developed nanoparticles, several experiments were performed. First of all, toxicity was evaluated by apoptosis determination after long-term exposure to nanoparticles. MDA-MB-231 and 4T1 cells were treated with different concentrations for 72 h, and apoptosis was determined by flow cytometry. Only the highest concentration of MSN-PEI-miR200c-HA induced a mild effect after 72 h of treatment in MDA-MB-231 cells, thus confirming the safety of the synthesized nanomaterials (Figure 2B, Supporting Figure S6).

Furthermore, the interactions of the cationic nanoparticles with negatively charged membranes were assessed by hemolysis experiments. Hemoglobin release from red blood cells was used to determine the potential toxicity of MSN-PEI-miR200c-HA. Our results demonstrate that nanoparticles do not have hemolytic activity, which indicates that MSN-PEI-miR200c-HA does not damage the red blood cell membranes (Figure 2C, Supporting Figure S7).

### Nanoparticles Target CD44 Receptor and Efficiently Deliver miR-200c *In Vitro*

To test the ability of our nanodevices to target the CD44 receptor, we compared the internalization of the nanoparticles by MDA-MB-231 cells, with a high expression of CD44, with the uptake by OE19 cancer cells, which have a negligible expression of CD44 (Supporting Figure S8). To carry out these internalization experiments, nanoparticles similar to MSN-PEI-miR200c-HA, yet loaded with the fluorescent dye TRITC (MSN-TRITC-PEI-miR200c-HA) were synthesized. The cells were incubated with MSN-TRITC-PEI-miR200c-HA for 15 min and then fixed and visualized by confocal microscopy. The cytoplasmic fluorescence in MDA-MB-231 confirms the uptake of the nanoparticles, which is significantly lower in OE19 (26% of control, *p* < 0.0001) (Figure 2D,E). These results evidence the ability of the HA-containing nanoparticles to target CD44 and effectively uptake MDA-MB-231 cells.

**Figure 3:**
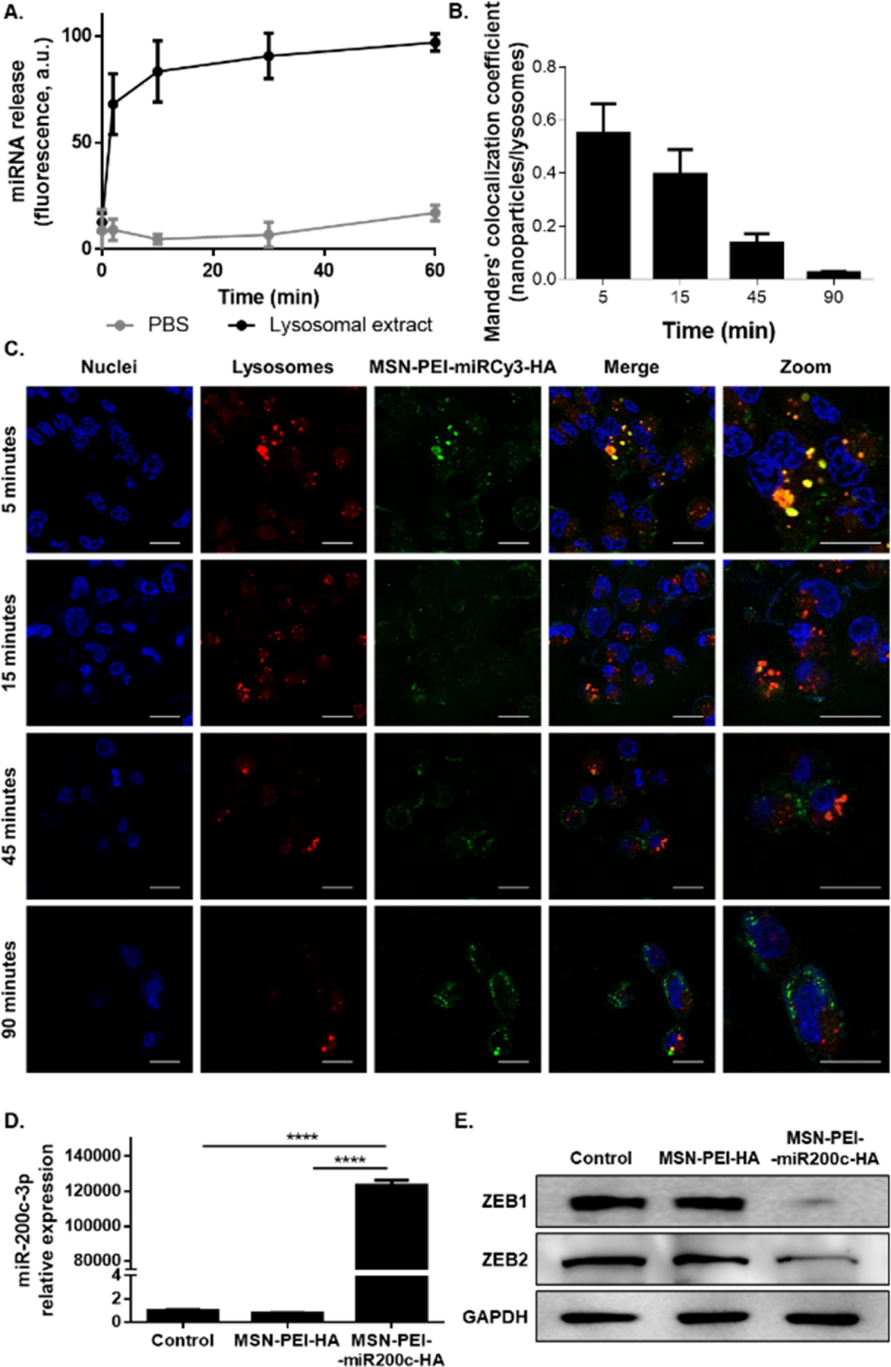
MSN-PEI-miR200c-HA efficiently delivered miR-200c-3p. (A) Release of miRCy3 from MSN-PEI-miRCy3-HA in PBS or lysosomal extract at 25 °C at the indicated time points (mean ± SD). (B, C) MDA-MB-231 was incubated with MSN-PEI-miRCy3-HA (green) for different time periods. Endosomes/lysosomes were labeled with LysoTracker Deep Red (red), and nuclei were stained with Hoechst33342 (blue). (B) Colocalization of nanoparticles and endosomes/lysosomes is shown in yellow color upon merging all fluorescent channels. Representative images. Scale bar: 20 μm. (C) Fraction of intracellular nanoparticles (MSN-PEI-miRCy3-HA) colocalizing with lysosomes. Manders colocalization coefficient is represented at several time points (mean ± SEM). (D) miR-200c-3p expression was determined by qRT-PCR in MDA-MB-231 cells untreated (control) and treated with MSN-PEI-HA or MSN-PEI-miR200c-HA (20 μg/mL) for 72 h. (E) Protein expression levels of ZEB1 and ZEB2 in MDA-MB-231 cells (control), and MDA-MB-231 cells treated with MSN-PEI-HA or MSN-PEI-miR-200c-HA (20 μg/mL) for 72 h. ****p < 0.0001.

It is crucial to ensure that synthesized nanoparticles are effective vectors to deliver miRNA into the cytoplasm. To evaluate this, similar nanoparticles to MSN-PEI-miR200c-HA but containing an RNA oligonucleotide labeled with Cy3 (miRCy3) were synthesized (MSN-PEI-miRCy3-HA). In this case, we found by fluorescence that the amount of labeled RNA not included in MSN-PEI-miRCy3-HA represented 0.7% of the total added, indicating that almost all of the miRNA added attached properly to the nanoparticles. CD44 targeting ability was confirmed (Supporting Figure S9), and the capability of nanoparticles to escape from lysosomes/endosomes was evaluated. MSN-PEI-miRCy3-HA was incubated in PBS or a lysosomal extract. There was no RNA release when nanoparticles were suspended in PBS, but a quick release of the labeled miRNA was observed when nanoparticles were suspended in lysosomal extract (Figure 3A). Besides, we also confirmed the release of the loaded miRNA in acetate buffer (pH = 5) (Supporting Figure S10). These results can be extrapolated to miR-200c-3p considering their similar chemistry and conformation. Furthermore, MDA-MB-231 cells were incubated with MSN-PEI-miRCy3-HA for several time periods (up to 90 min), and lysosomes and endosomes were labeled using LysoTracker Deep Red. Colocalization of nanoparticles and endosomes/lysosomes was detected after 5 min (Manders’ coefficient = 0.5500), demonstrating the rapid uptake of nanoparticles and their entrapment in endosomes/lysosomes after their internalization (Figure 3B,C). Noteworthy, by 45 min, a dispersion of miRNA throughout the cytosol was confirmed (Manders’ coefficient = 0.1356), thus confirming that our nanodevices allow the efficient delivery of miRNA in the cytosol. Moreover, we also assessed that the delivery of the miRNA by MSN-PEI-miR200c-HA in MDA-MB-231 cells upregulated the intracellular levels of miR-200c-3p (Figure 3D). Besides, we verified that treatment of MDA-MB-231 cells with MSN-PEI-miR200c-HA induced downregulation of ZEB1 and ZEB2 at the protein level, thus confirming the functionality of miR-200c-3p (Figure 3E).

### Anticancer Effect of Nanoparticles *In Vitro*

**Figure 4:**
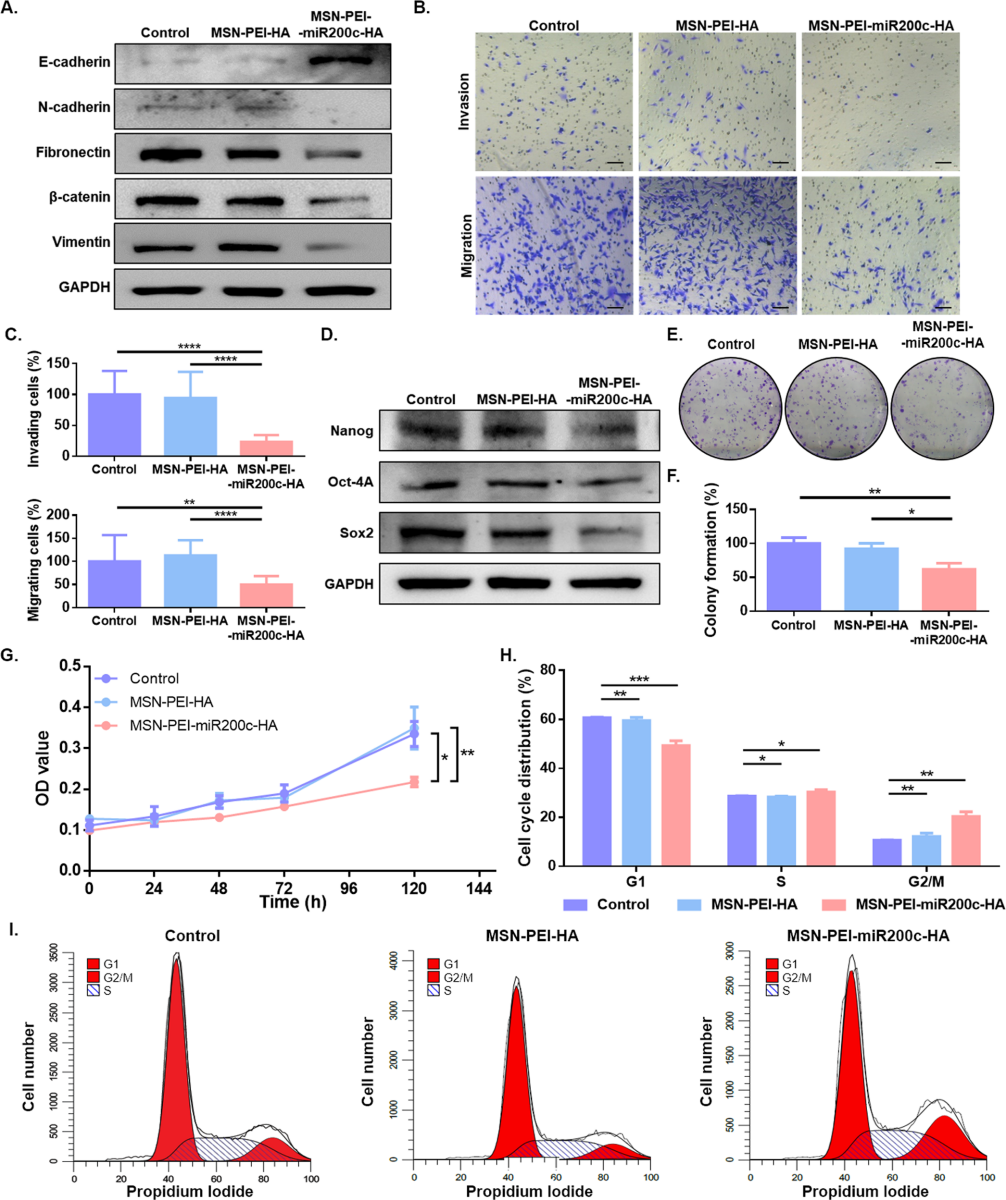
Effect of MSN-PEI-miR200c-HA on EMT, invasion, migration, stem-like properties, colony formation, and the cell cycle. MDA-MB-231 cells were treated with MSN-PEI-HA or MSN-PEI-miR-200c-HA for 72 h (20 μg/mL). Untreated cells (Control) were also included. (A) Protein expression levels of E-cadherin, N-cadherin, fibronectin, β-catenin, vimentin, and GAPDH. (B, C) Invasion and migration assays: Representative images (scale bar: 100 μm) (B) and quantification (mean ± SD) (C). (D) Protein expression levels of Nanog, Oct-4A, Sox2, and GAPDH. E-F. Colony formation assays: Representative images (E) and quantification (mean ± SD) (F). (G) Cell proliferation analysis (mean ± SD). (H, I) Cell cycle analysis by flow cytometry: Quantifications (mean ± SD) (H) and representative cell cycle profiles (I). OD: optical density; *p < 0.05; **p < 0.01; ***p < 0.001; ****p < 0.0001.

After the efficacy of MSN-PEI-miR200c-HA was confirmed to deliver the miRNA and to downregulate ZEB1 and ZEB2, its ability to inhibit EMT was assessed. Treatment with the miR-200c-3p-bearing nanoparticles increases protein expression of the epithelial marker E-cadherin and decreases expression of mesenchymal markers (N-cadherin, fibronectin, β-catenin, and vimentin) (Figure 4A). These results are in line with those obtained by Gregory et al., which demonstrated that ectopic expression of miR-200c reverts EMT.^63^ The nanoparticles also induce significant inhibition of migration and invasion capacity of MDA-MB-231 cells (Figure 4B,C).

Due to the fact that EMT confers stem cell-like properties,^9,10,64^ we further evaluated the expression of the stem cell markers Oct-4A, Nanog, and Sox2. As expected, expression levels of the BCSC markers are downregulated upon treatment with MSN-PEI-miR200c-HA (Figure 4D). Besides, cells treated with MSN-PEI-miR200c-HA show a reduced colony formation ability, thus confirming its potential against tumorigenesis (Figure 4E,F). No effect is observed when the control nanoparticles MSN-PEI-HA are used. These results evidence that MSN-PEI-miR200c-HA inhibits invasion and migration, stem-like properties, and tumorigenesis through reverting EMT.

Given that previous works demonstrated that miR-200c-3p inhibits tumor cell proliferation,^65−67^ we further studied the effect of nanoparticles on cell growth. Cell proliferation assays revealed that MSN-PEI-miR200c-HA significantly decreases tumor cell proliferation (Figure 4G) and induces cell cycle arrest in G2 (the percentage of cells in G1 decreased by 10%, and G2/M increased by 10%) (Figure 4H,I), confirming the antiproliferative effect of the MSN-PEI-miR200c-HA nanodevice. MDA-MB-231 transfected with miR-200c-3p was evaluated as a positive control to confirm that the observed effects are attributable to the presence of miRNA in the nanoparticles (Supporting Figure S11).

### *In Vivo* Therapeutic Efficacy

**Figure 5:**
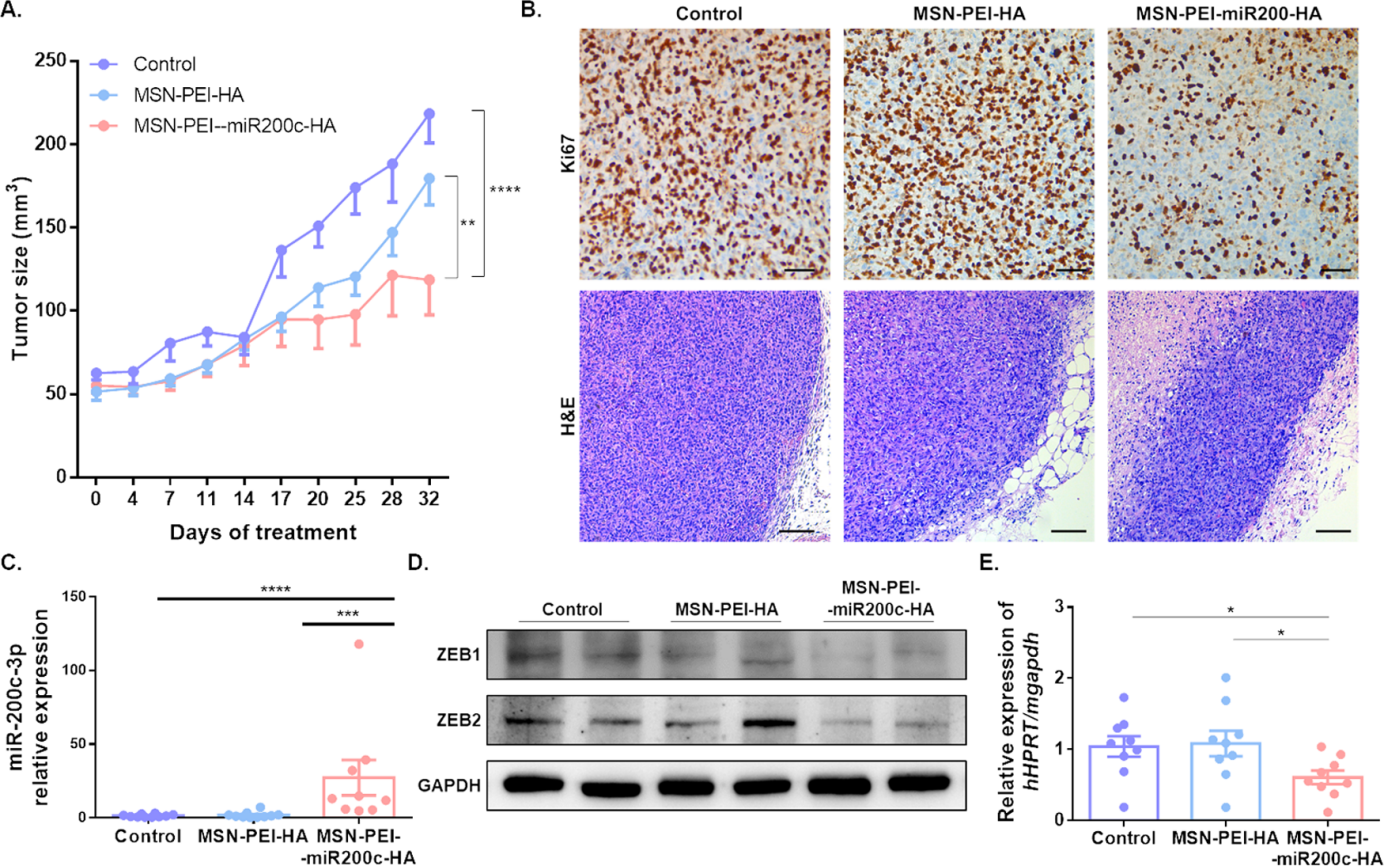
Antitumor activity of MSN-PEI-miR200c-HA *in vivo*. (A) Tumor growth rate in mice treated with PBS, MSN-PEI-HA, and MSN-PEI-miR200c-HA (mean ± SEM, *N* = 27). (B) Representative images of *K*_i_-67 (scale bar: 50 μm) and H&E (scale bar: 100 μm) staining of tumor sections. (C) miR-200c-3p expression in xenograft tumors determined by qRT-PCR (mean ± SEM). (D) Protein expression levels of ZEB1, ZEB2, and GAPDH in xenograft tumors. (E) *hHPRT* expression relative to *mGAPDH* in lung tissue was determined by qRT-PCR (mean ± SEM). *p < 0.05; **p < 0.01; ***p < 0.001; ****p < 0.0001.

Encouraged by the results presented above, we next assessed the potential of MSN-PEI-miR200c-HA for BC therapy *in vivo*. Mice bearing orthotopic tumors were treated with vehicle, MSN-PEI-HA, or MSN-PEI-miR200c-HA. Treatment with MSN-PEI-miR200c-HA significantly decreases the tumor size compared to the case with vehicle and MSN-PEI-HA (Figure 5A, Supporting Figure S12). Besides, the partial inhibitory effect found for control MSN-PEI-HA nanoparticles can be tentatively attributed to nanoparticles accumulation in tumors, which could induce a mild cytotoxic effect. Moreover, H&E and *K*_i_-67 staining of tumor sections after 28 days of treatment confirm the antiproliferative effect of MSN-PEI-miR200c-HA (Figure 5B), which is consistent with the cell cycle arrest observed *in vitro* (*vide ante*). Besides, 48 h after the last treatment with MSN-PEI-miR200c-HA, tumor and major organs were harvested, and biodistribution of nanoparticles was determined by Si quantification by ICP-MS and miR-200c-3p quantification by qRT-PCR. The results confirm the tumor-targeting ability of the nanoparticles, although nanoparticles are also found in lungs, spleen, kidney, and liver (Supporting Figure S13).

Having shown that MSN-PEI-miR200c-HA accumulate in tumors, their efficacy to modulate the expression of miR-200c-3p, and its targets ZEB1 and ZEB2 *in vivo*, was assessed. Upregulation of miR-200c-3p (Figure 5C) and downregulation of ZEB1 and ZEB2 (Figure 5D) are confirmed in tumors, which evidence the efficient delivery of functional miR-200c-3p *in vivo*. Moreover, it should be noted that a lower expression of *hHPRT* in the lungs of mice treated with MSN-PEI-miR200c-HA compared to control groups indicates that miR-200c-3p inhibits lung metastasis (Figure 5E), which was confirmed by visualizing lung sections (Supporting Figure S14).

Renal toxicity and hepatotoxicity were evaluated by determining the plasma levels of CRE, URE, AST, and ALT in untreated mice and mice treated with MSN-PEI-miR200c-HA or MSN-PEI-HA, yet no significant differences in these markers are observed (Supporting Figure S15). Besides, there are no differences in body weight among groups (Supporting Figure S16). These results show that nanoparticles are well tolerated. Taken together, these data strongly suggest that MSN-PEI-miR200c-HA is an effective and safe carrier for the targeted and controlled delivery of miR-200c-3p in tumors for BC treatment.

## Conclusions

In summary, we have developed MSNs containing PEI, miR-200c-3p, and HA (MSN-PEI-miR200c-HA) for the *in vivo* delivery of miR-200c-3p as BC therapy. Results from *in vitro* studies demonstrate the biocompatibility of MSN-PEI-miR200c-HA, their ability to target the CD44 receptor, and their capacity to escape from endosomes/lysosomes, which is crucial to ensuring the delivery of miRNA into cytosol. Besides, the delivery of miR-200c-3p reverts EMT, invasion, migration, stem-like phenotype, tumorigenic ability, and proliferation of tumor cells. The nanoparticles were also tested *in vivo* in a TNBC orthotopic xenograft mouse model, which exhibited a remarkable effect on tumor growth and metastasis inhibition as a result of the effective delivery of miR-200c-3p in the tumor without any relevant toxicity. Moreover, biodistribution studies confirm a significant accumulation of nanoparticles in tumors. Overall, our data suggest that these nanodispositives have potential clinical relevance for the treatment of BC. Furthermore, our findings point toward the potential of MSNs as a promising approach for the systemic delivery of miRNAs for BC treatment and encourage us to continue exploring their applicability for combinatorial treatments based on the delivery of miRNAs and drugs used in clinical practice.

## Data Availability

The raw data required to reproduce these findings are available from the authors upon request.

## Supporting Information Available

X-ray diffraction pattern of MSNs before and after calcination; nitrogen adsorption–desorption isotherms of MSN scaffold; TEM images of MSNs; 4T1 cells were treated with MSN-PEI-miR200c-HA at different concentrations; CD44 targeting ability of MSN-PEI-miRCy3-HA; organ distribution analysis of MSN-PEI-miR200c-HA; and changes in body weight of xenograft-bearing mice (PDF)
