# Supplementary material for: Delivery of miR-200c-3p Using Tumor-Targeted Mesoporous Silica Nanoparticles for Breast Cancer Therapy
Source: ACS Appl Mater Interfaces. 2023 Aug 7;15(32):38323–34. doi: 10.1021/acsami.3c07541 (PMC10436244; doi:10.1021/acsami.3c07541)
Supplement: Supplementary file 1 — am3c07541_si_001.pdf [file am3c07541_si_001.pdf]

Supporting Information

Delivery of miR-200c-3p Using Tumor-Targeted Mesoporous Silica Nanoparticles for Breast Cancer Therapy

Iris Garrido-Cano[a,b], Anna Adam-Artigues[a], Ana Lameirinhas[a], Juan F. Blandez[b,c,d], Vicente Candela-Noguera[b], Ana Lluch[a,e,f,g], Begoña Bermejo[a,e,g], Felix Sancenón[b,c,d,h], Juan Miguel Cejalvo[a,e,g*], Ramón Martínez-Máñez[b,c,d,h*], Pilar Eroles[a,e,f*]

[a]Biomedical Research Institute INCLIVA, Valencia, 46010, Spain. Igarrido@incliva.es

[b]Instituto Interuniversitario de Investigación de Reconocimiento Molecular y Desarrollo Tecnológico (IDM), Universitat Politècnica de València, Universitat de València, Valencia, 46010, Spain.

[c]CIBER de Bioingeniería, Biomateriales y Nanomedicina (CIBER-BBN), Madrid, 28029, Spain.

[d]Unidad Mixta de Investigación en Nanomedicina y Sensores. Universitat Politècnica de València, IIS La Fe, Valencia, 46026, Spain

[e]Centro de Investigación Biomédica en Red de Cáncer (CIBERONC), Madrid, 28029, Spain.

[f]Universitat de València, Valencia, 46010, Spain

[g]Clinical Oncology Department, Hospital Clínico Universitario de Valencia, Valencia, 46010, Spain.

[h]Unidad Mixta UPV-CIPF de Investigación en Mecanismos de Enfermedades y Nanomedicina. Universitat Politècnica de Valencia, Centro de Investigación Príncipe Felipe, Valencia, 46012, Spain.

*Correspondence should be addressed to J. M. C.: jmcejalvo@incliva.es (phone number: +34 961973517), R. M.-M.: rmaez@qim.upv.es (phone number: +34 963877343), and P. E.: pilar.eroles@uv.es (phone number: +34 963864100)

SUPPLEMENTAL FIGURES

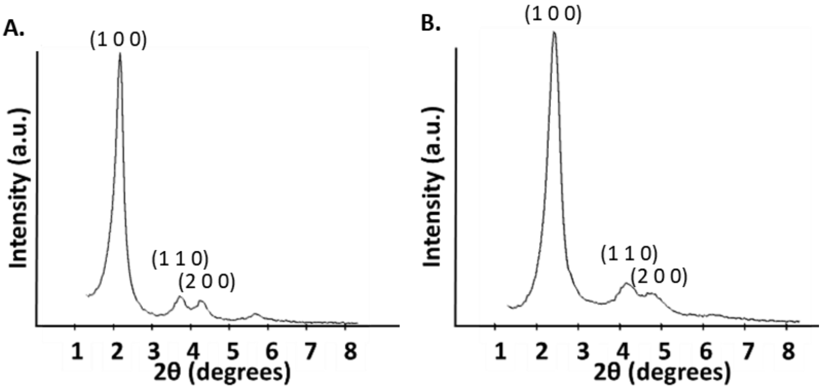

Figure S1. X-ray diffraction pattern of MSNs before (A) and after (B) calcination. Typical patterns of a hexagonal-ordered pore array that can be indexed as (100), (110), and (200) Bragg reflections.

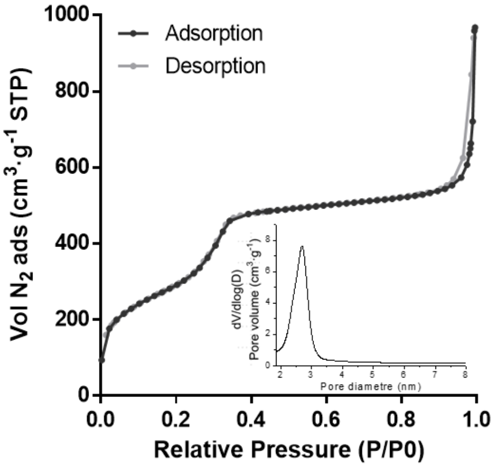

Figure S2. Nitrogen adsorption−desorption isotherms of MSN scaffold (inset: pore-size distribution).

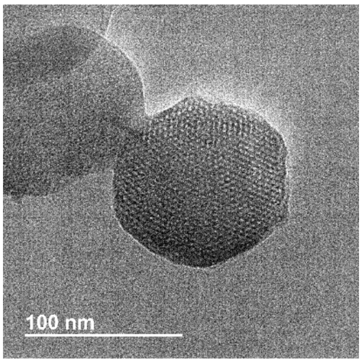

Figure S3. TEM images of MSNs.

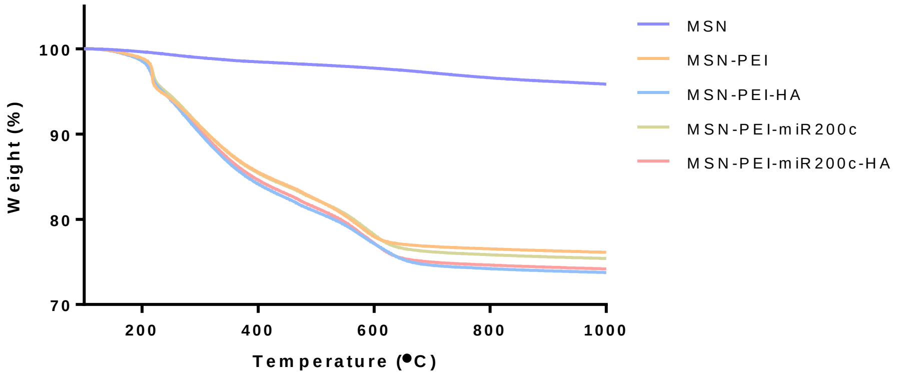

Figure S4. Thermogravimetric analysis of MSN scaffold, MSN-PEI, MSN-PEI-HA, MSN-PEI-miR200c, and MSN-PEI-miR-200c-HA. Calcined MSNs showed a 4.1% of weight loss, which can be assigned to silanol groups condensation. Considering the functionalized nanoparticles have an equivalent amount of silanol groups, the organic content calculated for MSN-PEI, MSN-PEI-HA, MSN-PEI-miR and MSN-PEI-miR-HA is 19.7, 22.1, 20.4 and 21.7, respectively. That means that PEI represents approximately the 19.7% of weight in the nanoparticles, which is around the 50% of the PEI that was added in the synthesis procedure to the nanoparticles (68 mg of PEI per 100 mg of MSN). The addition of HA to MSN-PEI increased the organic content a 2.4%, which can be attributed to the amount of HA in MSN-PEI-HA and fits with the amount of HA added in the procedure (2.37 mg of HA per 100 mg of MSN). Regarding the addition of miR-200, the MSN-PEI-miR have a 0.7% of additional organic content, which is attributable to the miR-200 attached to the nanoparticles and also fits with the amount of miR-200 added in the procedure (5 nmol or 0.732 mg of miR per 100 mg of MSN). In the case of MSN-PEI-miR-HA, the organic content is lower than the sum of PEI, miR and HA fractions separately (19.7 + 0.7 + 2.4 = 22.8% > 21.7%). This can be explained as the attachment of miR in the surface through electrostatic bonds can hinder some interactions of HA with positive charges of PEI. Hence, considering that the amount of PEI and miR is equivalent in MSN-PEI-miR-HA and MSN-PEI-miR, the weight attributed to HA can be estimated as ca. 1.3%, which is ca. the 55% of HA added in the procedure. All the data were calculated by taking the dry weight, that is, considering the weight loss up to 100 °C was due to the nanoparticles moisture.

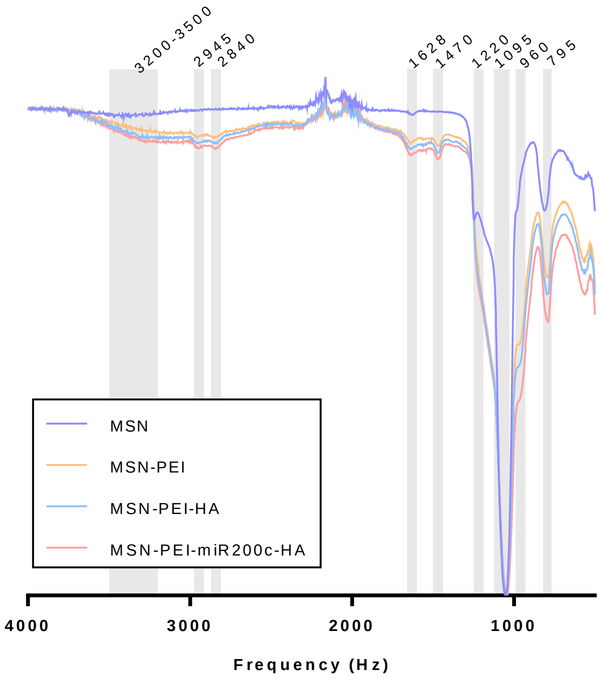

Figure S5. Attenuated total reflectance (ATR) spectra of MSN scaffold, MSN-PEI, MSN-PEI-HA, and MSN-PEI-miR-200c-HA. All the materials present the typical peaks at 1095 and 1220 cm^{-1}, corresponding to bending of Si-O-Si group, 795 cm^{-1} to Si-O stretching, and 960 cm^{-1} to Si-OH bending. After coating with PEI different peaks appear, such as a characteristic amine group broad band (3400-3200 cm^{-1}, NH stretching), two peaks at 2840 and 2945 cm^{-1} corresponding to CH_2 and CH_3 vibrations, and two peaks centered at 1628 and 1470 cm^{-1} that correspond with the NH_2 deformation and C-H scissoring, respectively, and a shoulder around 1220 cm^{-1} corresponding to C-N stretching. After the addition of HA, the signals corresponding to N-H, C-H, and C-N are intensified, and specifically the corresponding with O-H stretching (3200-3400 cm^{-1}). The presence of miR-200c-3p in MSN-PEI-miR-200c-HA implies the increase in 960 cm-1, which can be attributed specifically to RNA.

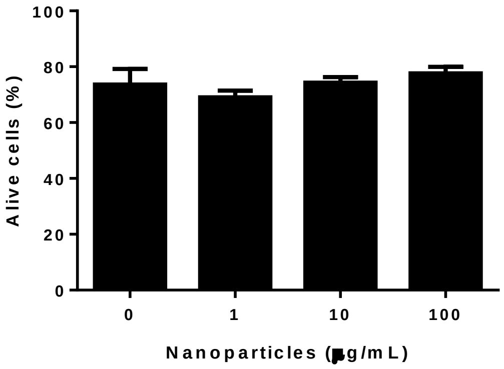

Figure S6: 4T1 cells were treated with MSN-PEI-miR200c-HA at different concentrations for 72 hours, and evaluated for apoptosis by flow cytometry after PI and FITC-Annexin V staining. Untreated cells were included as a control (Mean ± SD).

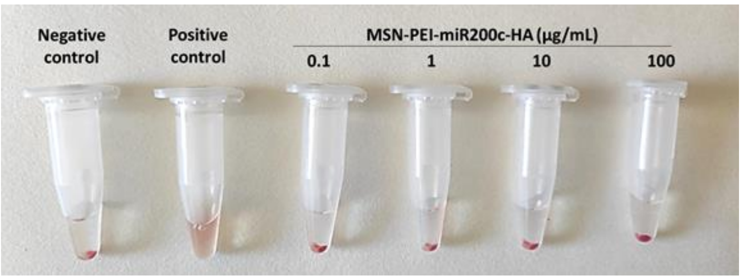

Figure S7. Hemolytic activity of MSN-PEI-miR200c-HA. Red blood cells were incubated with PBS (negative control), 1% Triton X-100 (positive control), or MSN-PEI-miR200c-HA at different concentrations for 1 hour at 37 °C. Representative images after centrifugation at 500 xg for 5 minutes.

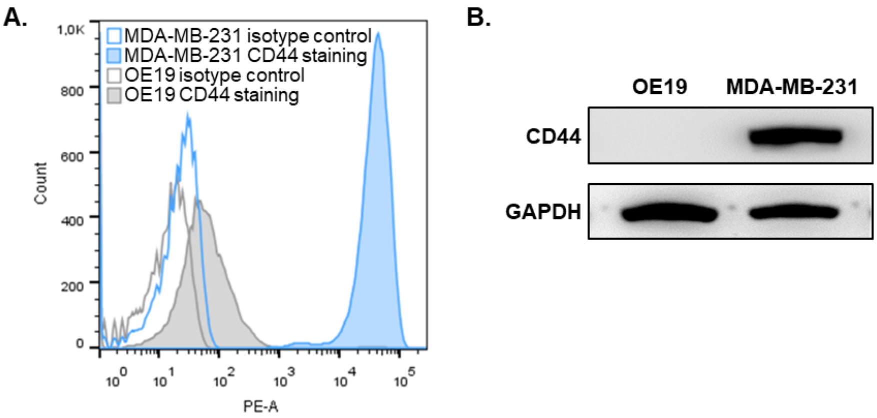

Figure S8. CD44 expression determined in OE19 and MDA-MB-231. A. Flow cytometry results. Histograms of CD44 (filled) and isotype control (empty) stained OE19 (grey) and MDA-MB-231 (blue) cells. B. Western blot results.

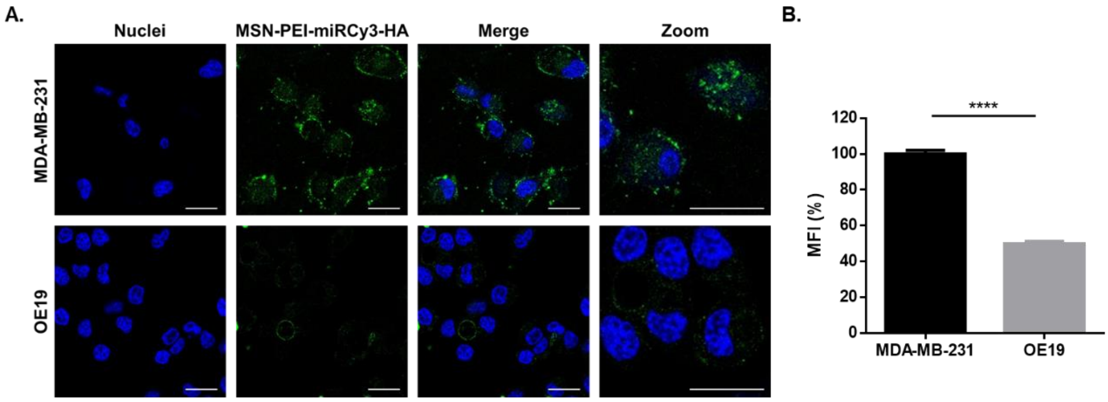

Figure S9. CD44 targeting ability of MSN-PEI-miRCy3-HA. Internalization of MSN-PEI-miRCy3-HA in MDA-MB-231 and OE19 cell lines after 25 minutes of exposure to nanoparticles (25µg/mL). Mean red fluorescent intensity (MFI, mean ±S EM) (A) and representative confocal images (scale bar: 20 µm) (B). **** $p < 0.0001$

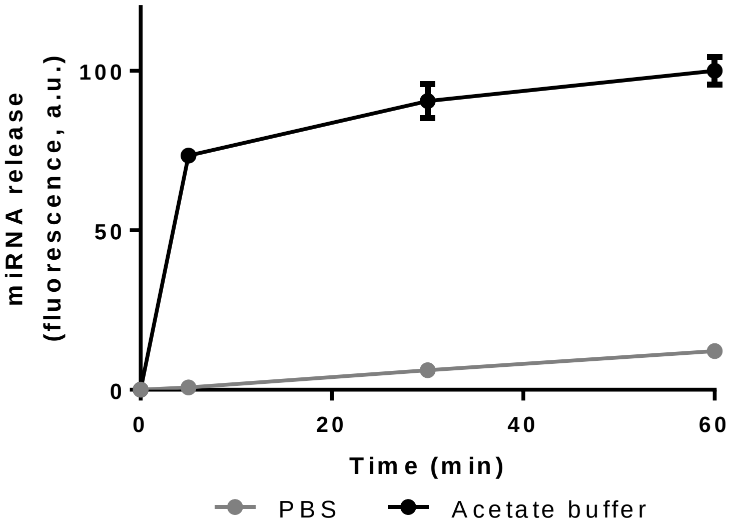

Figure S10. MSN-PEI-miR200c-HA efficiently deliver miR-200c-3p. Release of miRCy3 from MSN-PEI-miRCy3-HA in PBS (pH 7.4) (grey) or acetate buffer (pH 5) (black) at 25°C at the indicated time points (mean ± SD).

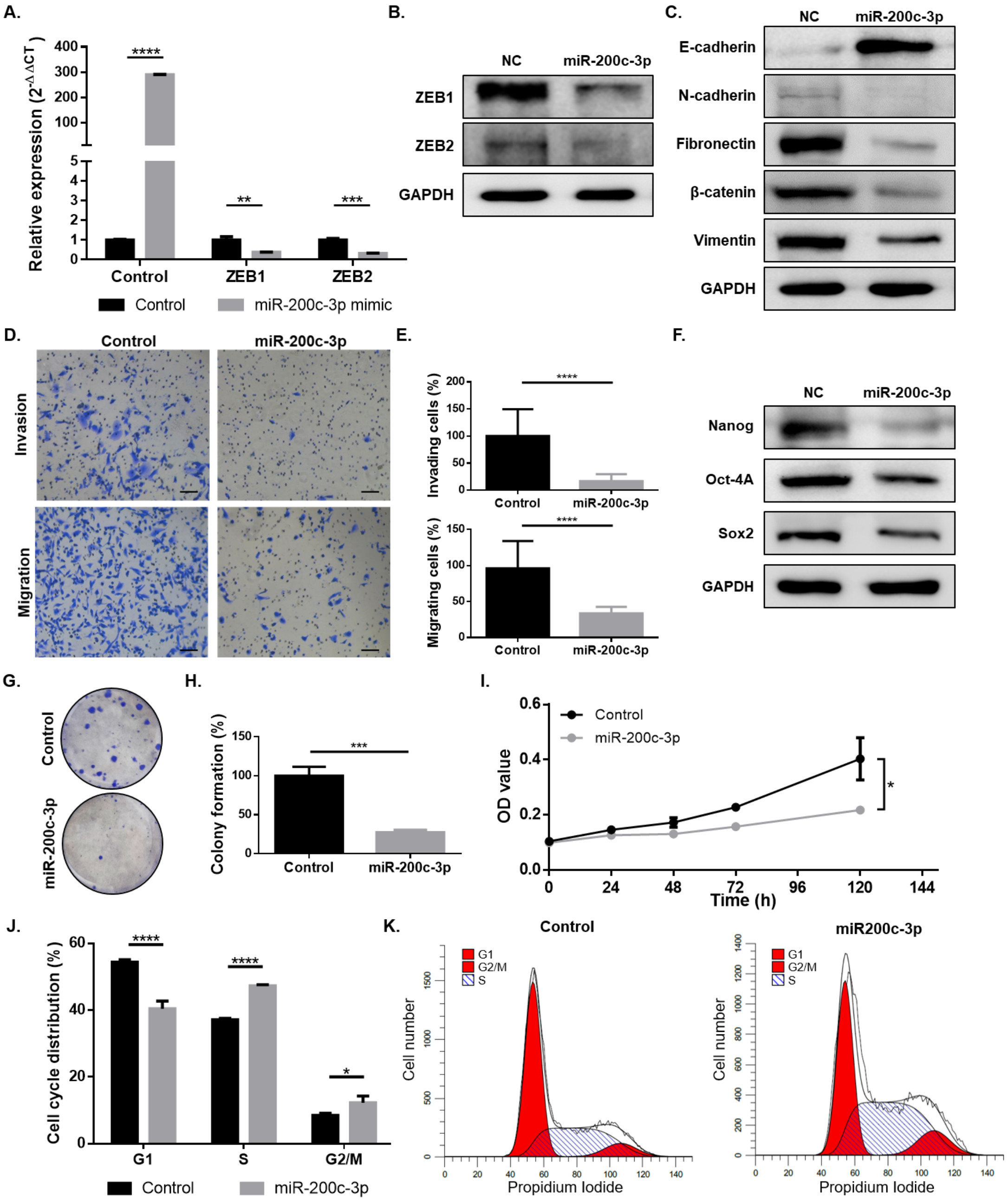

Figure S11. Effect of miR-200c-3p on EMT, invasion, migration, stem-like properties, colony formation, and cell cycle. MDA-MB-231 cells were transfected with scramble miRNA (Control) or miR-200c-3p and evaluated after 72 hours. A. miR-200c-3p, ZEB1 and ZEB2 expression was determined by qRT-PCR. B. Protein expression levels of ZEB1 and ZEB2. C. Protein expression levels of E-cadherin, N-cadherin, fibronectin, β-catenin, vimentin, and GAPDH. D-E. Invasion and migration assays: Representative images (Scale bar: 100 µm) (D) and quantification (mean ± SD) (E). F. Protein expression levels of Nanog, Oct-4A, Sox2, and GAPDH. G-H. Colony formation assays. Representative images (G) and quantification (mean ± SD) (H). I. Cell proliferation analysis (mean ± SD). J-K. Cell cycle analysis by flow cytometry. Quantifications (mean ± SD) (J) and representative cell cycle profiles (K). OD: optical density; *: p <0.05; **: p <0.01; ***: p <0.001; ****: p <0.0001.

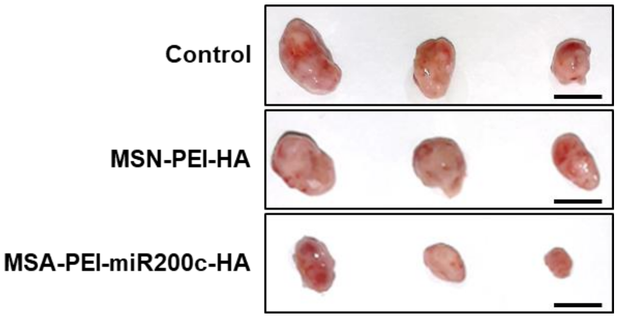

Figure S12: Representative images of *ex vivo* tumors. Scale bar: 1 cm.

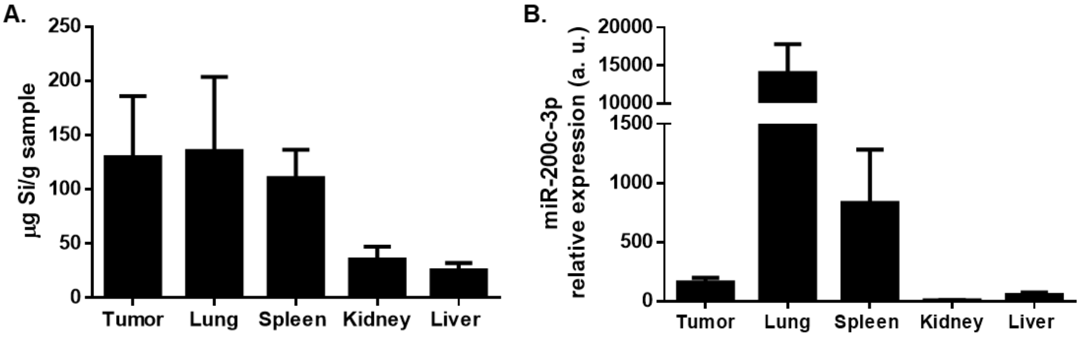

Figure S13. Organ distribution analysis of MSN-PEI-miR200c-HA. A: Si biodistribution detrmined by ICP-MS. B: miR-200c-3p biodistribution by qRT-PCR.

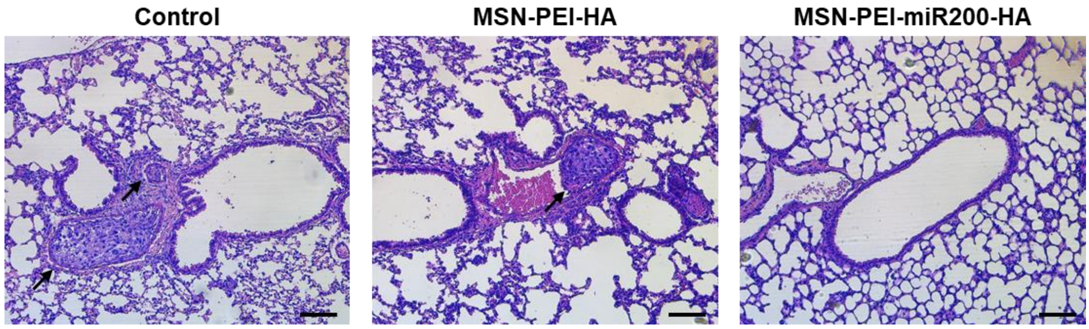

Figure S14. Representative images of H&E staining of lung sections. Arrows indicate lung metastases. Scale bar: 100 um.

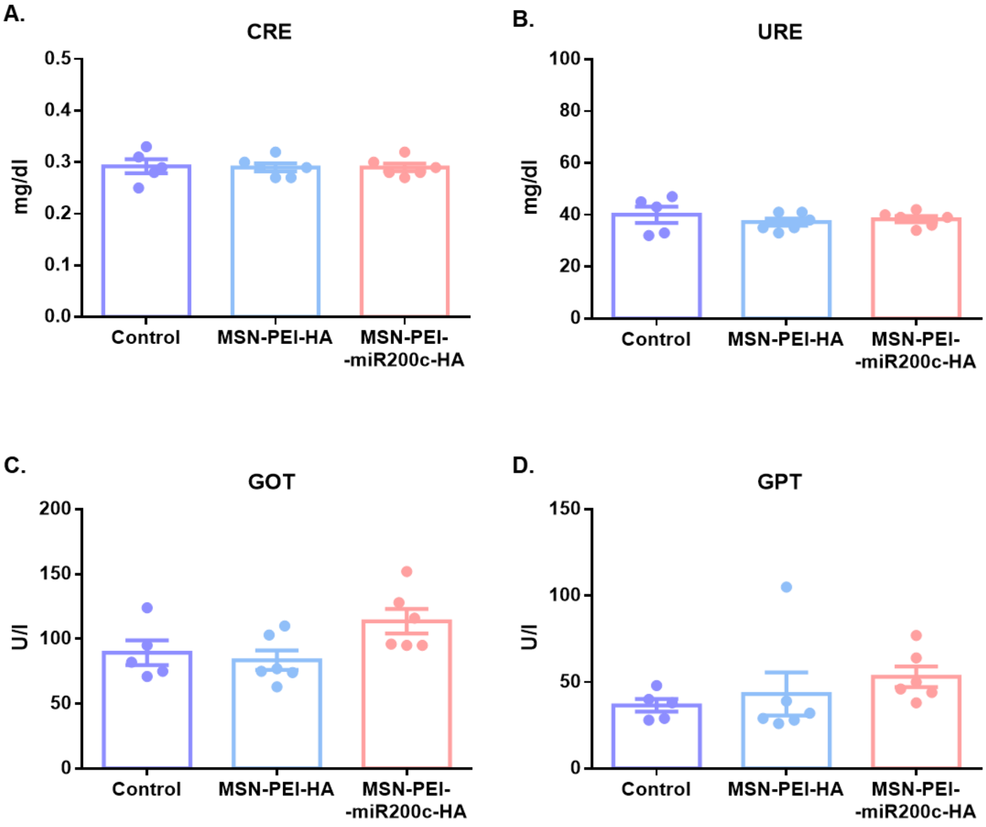

Figure S15. Serum levels of CRE (A), URE (B), AST (C), and ALT (D) are in the normal range 48 hours after the last dose of treatment. mean ± SEM.

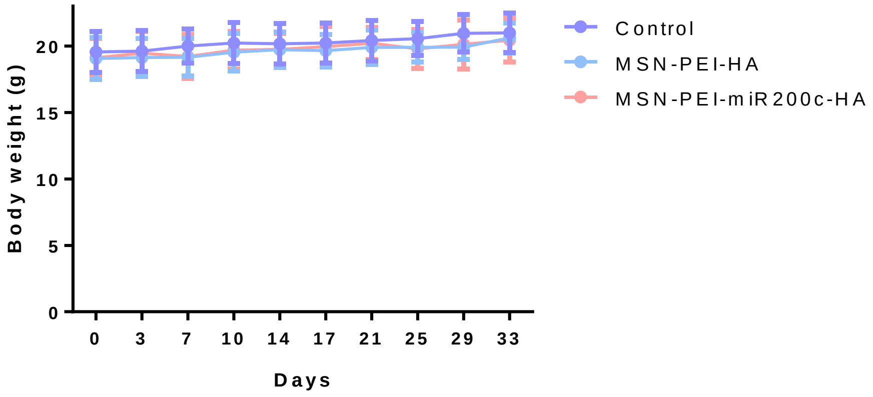

Figure S16. Changes in body weight of xenograft-bearing mice. Mean ± SD.
